# Phytochemical Constituents and Biological Activities of *Melicope lunu-ankenda*

**DOI:** 10.3390/molecules23102708

**Published:** 2018-10-20

**Authors:** Enas Mohamed Eliaser, Jun Hui Ho, Najihah Mohd. Hashim, Yaya Rukayadi, Gwendoline Cheng Lian Ee, Ahmad Faizal Abdull Razis

**Affiliations:** 1 UPM-MAKNA Cancer Research Laboratory, Institute of Bioscience, Universiti Putra Malaysia, 43400 UPM Serdang, Selangor, Malaysia; en_mo2008@yahoo.com; 2 Unit of Genetics and Molecular Biology, Institute of Biological Sciences, Faculty of Science, University of Malaya, Jalan Universiti, 50603 Kuala Lumpur, Malaysia; winxonho@gmail.com; 3 Pharmacy Department, Faculty of Medicine, University of Malaya, 50603 Kuala Lumpur, Malaysia; najihahmh@um.edu.my; 4 Center of Natural Product Research and Drug Discovery (CENAR), University of Malaya, 50603 Kuala Lumpur, Malaysia; 5 Department of Food Science, Faculty of Food Science and Technology, Universiti Putra Malaysia, 43400 UPM Serdang, Selangor, Malaysia; yaya_rukayadi@upm.edu.my; 6 Chemistry Department, Faculty of Science, Universiti Putra Malaysia, 43400 UPM Serdang, Selangor, Malaysia; gwendolinechenglian@gmail.com; 7 Laboratory of Molecular Biomedicine, Institute of Bioscience, Universiti Putra Malaysia, 43400 UPM Serdang, Selangor, Malaysia; 8 Laboratory of Food Safety and Food Integrity, Institute of Tropical Agriculture and Food Security, Universiti Putra Malaysia, 43400 UPM Serdang, Selangor, Malaysia

**Keywords:** *Melicope lunu-ankenda*, *Rutaceae*, phytochemical, biological activities, ethnopharmacognosy

## Abstract

Natural products, either pure compounds or standardized plant extracts, have provided opportunities for the discovery of new drugs. Nowadays, most of the world’s population still relies on traditional medicines for healthcare purposes. Plants, in particular, are always used as traditional medicine, as they contain a diverse number of phytochemicals that can be used for the treatment of diseases. The multicomponent feature in the plants is considered a positive phytotherapeutic hallmark. Hence, ethnopharmacognosy has been the focus for finding alternative treatments for diseases. *Melicope lunu-ankenda*, also known as *Euodia lunu-ankenda*, is widely distributed in tropical regions of Asia. Different parts of *M. lunu-ankenda* have been used for treatment of hypertension, menstrual disorder, diabetes, and fever, and as an emmenagogue and tonic. It has also been consumed as salad and as a condiment for food flavorings. The justification of use of *M. lunu-ankenda* in folk medicines is supported by its reported biological activities, including its cytotoxic, antibacterial, antioxidant, analgesic, antidiabetic, and anti-inflammatory activities. This review summarizes the phytochemical compounds isolated from various parts of *M. lunu-ankenda*, such as root and leaves, and also its biological activities, which could make the species a new therapeutic agent for some diseases, including diabetes, in the future.

## 1. Introduction

In the last few decades, herbal drugs have gained increased popularity in the treatment of ailments. The unparalleled availability of chemical diversity of herbal products allows them to provide limitless opportunities for the discovery of new drugs. In Asia, the use of traditional medicines derived from herbs represents a long history of interactions between humans and their environments. Plants that have been used for the production of traditional medicines are found to contain many different phytochemical compounds that can be used for the treatment of chronic and infectious diseases [1]. Due to increasing microbial resistance and the undesirable side effects of chemically synthesized drugs, the use of herbs to make natural drugs has become topical again [1,2]. Many phytochemical compounds have been found to be safe and effective with lower adverse effects than chemically synthesized drugs, and their beneficial biological activities such as anticancer, antimicrobial, antioxidant, antidiarrheal, analgesic, and wound-healing activities have been reported [1]. Under many circumstances, people have claimed the benefits of certain natural or herbal products. Nonetheless, clinical trials need to be conducted to demonstrate the effectiveness of bioactive compounds to verify the claim. Besides, a careful evaluation is required for the trials that are directed to understand the pharmacokinetics, bioavailability, efficacy, safety, and drug interactions of new bioactive compounds and their formulations [1]. This review focuses on *Melicope lunu-ankenda*, which is well-known for its medicinal application in folk medicine tradition. A lot of studies have reported on the phytochemical compounds that have been isolated from different parts of the plant such as its root, leaves, and stem, and biological activities, which both will be described in this review.

### 1.1. The Family Rutaceae

*M. lunu-ankenda* is a species belonging to the family *Rutaceae*. The leaves are pinnate, trifoliolate, or unifoliolate but less commonly simple; and alternate or spirally arranged, opposite, whorled (rare), and are frequently dotted with oil glands, which appear as dark green spots beneath surface while as translucent spots when they are exposed to light [3,4]. The inflorescences are either terminal or axillary panicles, cymes, racemes, or solitary (uncommon) and epiphyllous (rare). The flowers are bisexual or unisexual, and regular or irregular (rare). Seeds are usually ovoid to ellipsoid shape or oblong, and with or without endosperm [3]. In addition, spines and thorns are occasionally found on their twigs and branches, and an aromatic or lime-like scent from broken twigs, fruits, or crushed leaves enables them to be easily recognized [4].

This family consists of about 160 genera and 1650 species. The species are widely distributed and are found primarily in tropical and subtropical regions. In Malaysia, particularly in Sabah and Sarawak, 23 genera and 75 species are found, of which 17 genera and 43 species are native trees and shrubs [3]. Those native species exist in various habitats, ranging from hills and mountains, lowlands, coastal areas, and offshore islands. Most of the species are found below 1300 m elevation, but a few of them can grow to higher altitudes such as *Citrus* and *Tetractomia* to 1800 m, and *Melicope* to 2400 m. The species perpetuate by dispersing fruits and seeds through birds (*Melicope*, *Zanthoxylum*), wind (*Tetractomia*), water (*Merope*), and fruit and seed-eating animals (*Citrus*, *Clausena*, *Glycosmis*, *Micromelum*, *Severinia*, and most other Aurantioideae). The species in this family are used for various different purposes, such as food sources, flavorings, traditional medicines, edible fruits, timber for construction, and ornamentals [3].

### 1.2. The Genus Melicope

In the genus *Melicope*, the leaves are opposite or whorled, and trifoliolate, unifoliolate, or simple. The inflorescences are axillary, lateral, or terminal (rare). The flowers are bisexual or unisexual and 4-merous. The number of stamens can be the same or twice the number of petals, and carpels are 2-ovulate. The seeds are shiny, smooth, and remain attached in dehisced fruits [5].

*Melicope* species are distributed in tropical regions, including Mascarene Island, the Hawaiian Islands, Australia, New Zealand, East and South Asia, and Polynesia [3,4]. It contains about 230 species, of which about 14 of them are found in Sabah and Sarawak, while another 10 species are distributed on the Malayan Peninsula [3,6].

*Melicope* has similar characteristics with other two closely related genera in the same family, *Tetradium* Lour. and *Euodia* J. R. Forst. and G. Forst. Previously, *Tetradium* was treated as a section of *Euodia* by a German botanist, Heinrich Gustav Adolf Engler, in his standard major work on the southeast Asian Pacific *Rutaceae*. Then, Thomas Gordon Hartley [5] transferred many species from *Euodia* to *Melicope* J. R. and G. Forst. The main morphological differences between *Euodia*, *Tetradium,* and *Melicope* are given in Table 1.

*M. lunu-ankenda*, which is also known as *Evodia lunu-ankenda* (Gaertn.) Merr., is one of the species in the genus *Melicope*. It is most closely related to *M. glabra* and is mainly distributed in tropical regions of Asia, from Himalayan southward to Sri Lanka, Indonesia, Philippines, Malaysia, and eastward to Thailand and Indochina [6,7,8,9]. It is mainly distributed in primary and secondary evergreen forest, but can be found in mixed-deciduous and deciduous forests, swamp forest, and cloud forest as well. In addition, it is mostly found at the elevation of about 1000 to 2000 m [7]. According to the IUCN Red List of Threatened Species 2017, assessed by Barstow and Rehel [9], *M. lunu-ankenda* is currently categorized as Least Concern (LC) due to its wide geographical distribution, but the threats from timber harvesting may reduce its population size.

### 1.3. Taxonomy of Melicope lunu-ankenda

According to Hartley [7], the name of *lunu-ankenda* is derived from Sinhalese language *lunu* (salt) and *ankenda* (horny fiber), referring to the plant’s occurrence at sea level and its cartilaginous endocarp. Besides, *ankenda* is also the Sinhalese name for *Acronychia pedunculata* (L.) Miq.; thus, *lunu-ankenda* can be translated as “sea level *Acronychia*”, since *A. pedunculata* has a cartilaginous endocarp as well.

According to Barstow and Rehel [9], the taxonomy of this species is as following in the Table 2.

Based on the information from Global Biodiversity Information Facility (GBIF) [10], the basionym of *M. lunu-ankenda* is *Fagara lunu-ankenda* Gaertn. Besides, there are synonyms for this plant species, such as:

- *Euodia lunu-ankenda* (Gaertn.) Merr.
- *Euodia roxburghiana* (Cham.) Benth.
- *Evodia lunu-ankenda* (Gaertn.) Merr.
- *Zanthoxylum aromaticum* (Blume) Miq.
- *Zanthoxylum roxburghianum* Cham.

*Euodia* is the original and correct spelling used for the synonym for *Melicope*, while *Evodia* is the orthographical variant of *Euodia* [6].

### 1.4. Morphology of Melicope lunu-ankenda

*M. lunu-ankenda* is a shrub or tree that can grow up to about 30 m tall. The leaves are opposite, trifoliolate, unifoliolate (uncommon) or quadri or pentafoliolate (rare), and leaflets are elliptic or obovate-shaped. The inflorescences are axillary, the flowers are unisexual and bisexual (rare), the sepals are round to ovate-triangular shaped, and the petals are ovate to elliptic. Next, the fruiting carpels that connate at the base are ellipsoid to obovoid in shape, and the shape of the seeds is round to ovoid or ellipsoid, but are compressed occasionally [3,7].

## 2. *Melicope* in Folk Usage

Many species from the genus *Melicope* have been used for decades in folk medicine traditions for healthcare purposes, including treatment for diseases and disorders. Here are some examples of *Melicope* species that have been used in folklore medicinal practices.

In China, the fruits of *Evodia rutaecarpa* have been used for the treatment of headaches, abdominal pain, postpartum hemorrhage, amenorrhea, and dysentery [11]. In Bangladesh, the raw stem of *M. anisata* is rubbed on the gum and teeth as toothpaste for oral hygiene [12].

*M. pteleifolia* (Champ. ex Benth) T.G. Hartley, also known as *Evodia lepta* (Spreng.) Merr., is a species that is widely distributed in Vietnam and China. In Vietnam, the extract from *M. pteleifolia*, which is called ‘*Ba chac*’, is used for the treatment of fevers, colds, and inflammatory conditions [13]. In Malaysia, it has been commonly used as a remedy for fever, emmenagogue, stomachache, rheumatism, wounds, and itches. In addition, it is used for lowering the blood pressure and preventing premature ejaculation [14].

*M. borbonica*, also known as *Euodia borbonica* var. *borbonica*, is a species that is endemic to Réunion Island, France. It has been used for wound-healing, blood-cleansing, and as a sudorific drug. It is also used for its aromatic properties and treatment of rheumatism [15].

In Papua New Guinea, the decoction prepared from the dried bark of *M. elleryana*, which is also known as *Euodia elleryana* F. Muell., is used for the treatment of malaria. The juice squeezed from its fresh bark is used as a contraceptive, whose effect is reported to last for two to three years. In addition, the dried leaves of *M. triphylla* (*Euodia anisodora*) are heated, and the sap is consumed to treat tuberculosis. Besides, the leaves are used with the leaves, bark, and fruit of *Citrus* for treating constipation, diarrhea, stomach pain, and removing intestinal worms [16].

*M. confusa* or its synonym as *Euodia confusa*, is mostly distributed in Borneo, the Philippines, and part of Indonesia. In the Philippines, its bark is used for treating spleen enlargement. In Taiwan, its root decoction or leafy shoot mixed with liquor is consumed to cure hives [3].

In Indonesia, the bark of *M. bonwickii* (*Euodia bonwickii*) is used in treating bites caused by leeches. The leaves of *M. latifolia* (*Euodia latifolia*) are used for the treatment of fever and cramps in Indonesia and peninsular Malaysia, and its resin is used as varnish and adhesive [3].

*M. madagascariensis* (*Evodia madagascariensis*) is the plant species endemic to Madagascar. Its crushed roots are used for wound-healing. The infusion of its stem bark is used to treat measles, and aromatic essential oil is distilled from its leaves for massages [17].

In Malaysia, *M. lunu-ankenda* is locally known as “tenggek burung” [18]. The leaves are consumed as salad and condiment for food flavoring, as well as traditionally used to revitalize the body [19,20,21]. The flowers are also being consumed to control the conditions of diseases and disorders, including hypertension, menstrual disorder, and fever [19,21]. The roots are used for the treatment of colds and rheumatism, and the timber is used in construction [19]. It is also used to treat diabetes. In India, different parts of the plant are used for treatment for fever, the improvement of complexion, and as an emmenagogue and tonic. The leaves are utilized for the treatment for diabetes in folklore medicinal practices [18]. In Eastern Ghats, one of the nine Floristic Zones in India, the decocted root is used to treat asthma and bronchitis by mixing with black pepper and salt [22].

## 3. Phytochemical Compounds Isolated from *Melicope lunu-ankenda*

According to Al-Zuaidy et al. [23], the leaf extract of *M. lunu-ankenda* is enriched with four major bioactive components, called isorhamnetin, skimmianine, scopoletin, and melicarpinone, which may attribute towards the antidiabetic and antioxidant activities in this species. *O*-prenylated flavonoid (3,5,4′-trihydroxy-8,3′-dimethoxy-7-(3-methylbut-2-enoxy) flavone), a molecule isolated from the leaves of *M. lunu-ankenda*, has been revealed to have antidiabetic activity [18]. Two quinoline alkaloids, buchapine and 3-(3-methyl-2-butenyl)-4-[(3-methyl-2-butenyl)oxy]-2(1*H*)-quinolinone, and three furoquinoline alkaloids, roxiamines A, B, and C were isolated from flowers, leaves, and twigs and it was revealed that only quinoline alkaloids possess anti-HIV inhibitory activity [24]. Ramli et al. [19] also found that the leaf extracts from *M. lunu-ankenda* contain mixtures of hydrocarbons and squalene, fatty acids, and esters. The same study revealed that dichloromethane and methanol from the crude extracts of leaves have very strong insecticidal activity. In addition, a *p*-coumaric acid derivative called *p*-*O*-geranylcoumaric acid or 3-{4-*O*-(3,7-dimethyl-2,6-octadienyl) phenyl}-2-propenoic acid, which is a geranylated coumaric acid, was isolated as the major compound of the leave extracts of *M. lunu-ankenda* [19].

Next, two major chromene-type compounds in the volatile oil and extracts, evodione and leptonol, have been shown to be biologically active. Leptonol demonstrated good antipyretic and antioxidant activities, while both chromenes showed moderate analgesic and anti-inflammatory activities, rationalizing the use of *M. lunu-ankenda* in treating conditions such as fever and inflammation in folklore practices [8]. Furoquinoline alkaloids, dictamnine, evolitrine, kokusaginine, and *N*-methyl-4-methoxy-2-quinolone, along with marmesin, were also successfully isolated from the basic fraction of bark extract [25]. Furthermore, dictamnine (4-methoxyfuro[2,3-b]quinoline) and evolitrine (4,7-dimethoxyfuro[2,3-*b*]quinoline), which were isolated from the bark of *M. lunu-ankenda*, were reported to have moderate antifeedant activity [26]. Lal et al. [27] found that evolitrine, which was isolated from the dichloromethane extract of *M. lunu-ankenda* twigs, and some of its derivatives (alkoxy, amino, and dihydro derivatives) were demonstrated to have anti-inflammatory activity. The flower oil of *M. lunu-ankenda* was found to contain mostly evodione, (E)-β-ocimene, isolycodolin, and alloevodionol, as well as have significant antibacterial activity [28]. Moreover, 8-acetyl-3,4-dihydroxy-5,7-dimethoxy-2,2-dimethylchroman, along with alloevodionol-7-methyl ether, 4-methoxy-1-methyl-2(1H)-quinolinone, evolitrine, isoevodionoland its methyl ether were isolated from ethanolic extracts of the aerial parts of *M. lunu-ankenda* [29].

In addition, Kumar et al. [30] isolated three phenylethanones (1-[2′,4′-dihydroxy-3′,5′-di(3″-methyl2″-butenyl)-6′-methoxy)]phenylethanone, 1-[2′,4′-dihydroxy-6′-(3″-methyl-2″-butenyloxy)-5′-(3″-methyl-2″-butenyl)]phenylethanone, and l-[2′,4′-dihydroxy-6′-(3″,7″-dimethylocta-2″,6″-dienyloxy)-5′-(3″-methyl-2″-butenyl)]phenylethanone), five furoquinoline alkaloids (dictamine, evolitrine, γ-fagarine, skimmianine, and kokusaginine), lupeol and bergapten from the root bark of *M. lunu-ankenda*. Besides, Ito et al. [31] isolated two new coumarins, melilunumarin A (7-(4-hydroxy-3-methylbutanoxy) coumarin) and melilunumarin B (7-(3-methyl-4-carboxybutanoxy) coumarin methyl ester), and a new benzaldehyde derivative, 3,4-dihydro-3,4-dihydroxy-2,2-dimethyl-2H-1-benzopyran-6-carboxaldehyde, named melilunumane, from *M. lunu-ankenda*. They also isolated 6-deoxyhaplopinol, marmesin, umbelliferone, kokusaginine, and evolitrine. Among them, melilunumarin A, 6-deoxyhaplopinol, and marmesin were shown to have inhibitory activity to Epstein-Barr virus (EBV), a herpesvirus known to be associated with cancers such as nasopharyngeal carcinoma (NPC) [31,32].

Table 3 shows the group, chemical structure, and biological activity of some of the phytochemical compounds isolated from *M. lunu-ankenda*.

## 4. Biological Activities of *Melicope lunu-ankenda*

The phytochemical compounds isolated from extracts of various parts of *M. lunu-ankenda* have been demonstrated to have many different biological activities including antioxidant, antidiabetic, analgesic, and antibacterial activities. Not only do these biological activities justify the use of the plant in folklore practices, they also indicate its great potential and economical value in food and health industries. Each biological activity of *M. lunu-ankenda* is described below.

### 4.1. Analgesic

Lalitha et al. [48] reported that the ethyl acetate extracts of the bark of *M. lunu-ankenda* showed analgesic activity at both 500 mg/kg and 1000 mg/kg concentrations, as the time Swiss albino mice reacted to pain stimulus was increased in both Eddy’s hot plate and heat conduction methods. Its activity is equipotent to standard analgesic drugs morphine sulfate, which was injected to mice at 5 mg/kg for comparison (standard), justifying the effectiveness and the use of this plant species as an analgesic drug. Besides, the extracts were non-toxic up to 3000 mg/kg body weight of the tested mice. However, the phytochemical profile of the extract is yet to be elucidated. Furthermore, evodione and leptonol were also proven to show moderate analgesic activity in acetic acid-induced writhing and tail immersion assays [8]. Both evodione and leptonol were administrated orally to mice at 50 mg/kg and 100 mg/kg for both assays, respectively. In the acetic acid-induced writhing assay, leptonol and evodione at 100 mg/kg demonstrated 53.6% and 56.7% inhibition respectively as compared to 75.6% inhibition showed by aspirin, which was used positive control in the assay. In tail immersion assay, the mice’s response times were 5.71 ± 0.04 s and 6.05 ± 0.05 s for leptonol and evodione respectively at 100 mg/kg, while aspirin (100 mg/kg) was 7.59 ± 0.07 s [8].

### 4.2. Anthelmintic

Venkatachalam et al. [49] found that both bark ethyl acetate and aqueous extracts of *M. lunu-ankenda* demonstrated anthelmintic activity in a dose-dependent manner (25 mg/mL, 50 mg/mL, and 100 mg/mL) in both *Eudrillus eugeniae* (earthworm) and *Ascaris lumbricoids* (roundworm), respectively. Besides, they found that the bark ethyl acetate extract of this plant species showed the most potent anthelmintic activity at 100 mg/mL among different doses applied to both earthworm and roundworm. The activity is comparable to the activity by standard drug Albendazole. Both earthworm and roundworm were used, because they have anatomical and physiological similarities with human intestinal earthworm and roundworm parasite. However, the phytochemical compounds that are responsible for the activity have yet to be determined.

### 4.3. Antibacterial

Quorum sensing (QS) is an ability that Gram-negative bacteria use to coordinate the population behavior, including virulence factors expression via extracellular signaling molecules, such as *N*-acyl-l-homoserine lactones (AHLs). QS involves the coupling of AHLs to a transcriptional activator that modulates QS-mediated gene expression [20]. According to Tan et al. [20], they found that *M. lunu-ankenda* has anti-QS properties, as its extracts inhibited the QS-dependent virulence determinants of human pathogens called *Pseudomonas aeruginosa* PAO1. However, the anti-QS compound in the extracts has yet to be identified, but it proved that endemic plants in Malaysia could become leads in searching for anti-QS compounds.

Sabulal et al. [28] reported that the flower oil of *M. lunu-ankenda* exhibited significant in vitro antibacterial activity against all of the tested Gram-negative and Gram-positive bacteria, especially against *Salmonella typhi* and *Klebsiella pneumoniae*, which are both Gram-negative bacteria. They found that the major constituents of the flower oil are evodione (38.9%), (E)-β-ocimene (12.4%), isolycodolin (11.7%), and alloevodionol (10.6%), which all take up more than 70% of the content of the oil. However, the mechanism of the antibacterial activity of the oil was not reported in this study.

Moreover, Manandhar et al. [29] found that among the compounds isolated from ethanolic extracts of *M. lunu-ankenda*, 8-acetyl-3,4-dihydroxy-5,7-dimethoxy-2,2-dimethylchroman, alloevodionol-7-methyl ether, evolitrine, 4-methoxy-1-methyl-2(1H) quinolinone and its isomer were revealed to demonstrate antibacterial activities against *Bacillus subtilis* and *Staphylococcus aureus*. The minimum inhibitory concentrations (MICs) for each of the compounds mentioned are 250 μg/mL, 250 μg/mL, 62.5 μg/mL, 250 μg/mL, and 250 μg/mL respectively. Nonetheless, the mechanisms or functions of those compounds that are involved in antibacterial activity were not reported as well.

### 4.4. Cytotoxic Activity/Chemopreventive Effect

AL-Zuaidy et al. [50] reported the cytotoxic potential of the leaf extract (60% ethanolic extract) of *M. lunu-ankenda* on 3t3-L1 (mouse preadipocytes) and HepG2 (hepatocellular carcinoma). Their results showed that the extract showed potent cytotoxic activity against HepG2 cells with IC_50_ of 20.33 ± 1.5 μg/mL and 13.7 ± 2.1 μg/mL after the exposure for 48 h and 72 h respectively by using MTT assay. On the other hand, the same extract was also found to be in minimal activity against 3t3-L1 cell with the IC_50_ of 143.7 ± 3.2 μg/mL, 93 ± 3 μg/mL, and 81 ± 2 μg/mL after exposure for three different respective time points: 24 h, 48 h, and 72 h. This shows that the leaf extract is cytotoxic to cancer cells, and leaves the normal, non-cancerous cells unaffected. Their findings also suggested the significance of *M. lunu-ankenda* as a medicinal plant and as an antiproliferative agent. They suggested that the cytotoxicity of the extract may be attributed to the phytochemicals, especially the alkaloids in the extract.

**Figure 1:**
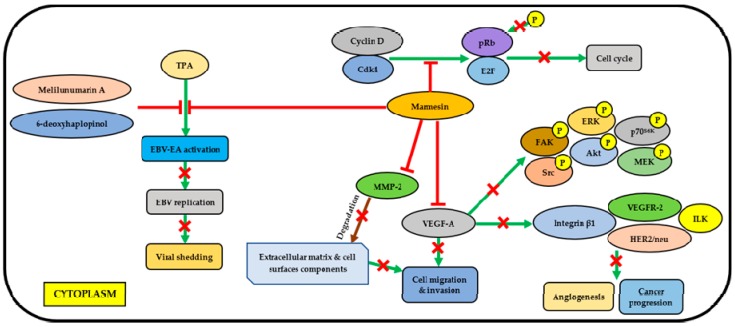
The mechanisms of cytotoxic/chemopreventive activity exhibited by marmesin. TPA, 12-*O*-tetradecanoylphorbol-13-acetate; EBV-EA, Epstein-Barr virus early antigen; Cdk, cyclin-dependent kinase; pRb, retinoblastoma protein; E2F, transcription factor E2F; P, phosphate; MMP-2, matrix metalloproteinase-2; VEGF-A, vascular endothelial growth factor A; FAK, focal adhesion kinase; Src, proto-oncogene tyrosine-protein kinase; ERK, extracellular signal-regulated kinase; Akt, protein kinase B; p70S6K, ribosomal protein S6 kinase beta-1; MEK, mitogen-activated protein kinase kinase; VEGFR-2, vascular endothelial growth factor receptor-2; ILK, integrin-linked kinase; HER2/neu, human epidermal growth factor receptor 2.

In addition, Ito et al. [31] found that three coumarins, melilunumarin A, 6-deoxyhaplopinol, and marmesin showed a significant chemopreventive effect on Epstein-Barr virus early antigen (EBV-EA) activation with IC_50_ of 303 mol ratio/TPA, 300 mol ratio/TPA. and 336 mol ratio/TPA respectively, by using a short-term in vitro assay of TPA-induced EBV-EA activation in Raji cells. These IC_50_ values are comparable to curcumin (IC_50_ 341 mol ratio/TPA) EBV is a γ-herpesvirus that is associated with human malignancies, such as Burkitt’s lymphoma (BL), nasopharyngeal carcinoma (NPC), and T-cell lymphoma [32]. Their findings suggested that the relative location of a hydroxy group and a hydrophobic prenyl moiety in coumarin may contribute to the chemopreventive effect of the compound against chemical-induced malignancy such as TPA [31]. Table 4 shows the functions or mechanisms of compounds contributing to the cytotoxic/chemopreventive activity of *M. lunu-ankenda*. Figure 1 demonstrates the mechanism of marmesin cytotoxic/chemopreventive activity.

Marmesin, melilunumarin A, and 6-deoxyhaplopinol were found to demonstrate inhibitory effect on TPA-induced EBV-EA activation in Raji cells [31]. Besides, marmesin inhibits pRb phosphorylation in human umbilical vein endothelial cells (HUVECs) and in non-small cell lung cancer (NSCLC) cell lines (A549 and H1299) by suppressing the expression of Cdk4 and cyclin D; hence, the cell cycle is halted [51,52]. It suppresses the expression and activity of MMPs (MMP-2) to prevent cell migration and invasion, which can also be prevented by inhibiting VEGF-A [51]. In addition, marmesin significantly inhibits the VEGF-A-induced phosphorylation of FAK, Src, ERK, Akt, p70^S6K^ and MEK, and expression of cellular signaling molecules including vascular endothelial growth factor receptor-2 (VEGFR-2), human epidermal growth factor receptor 2 (HER2/neu), integrin β1 and integrin-linked kinase (ILK), which are closely associated with angiogenesis and cancer progression [51,52].

### 4.5. Antidiabetic

It is known that diabetes mellitus (DM) is the result of oxidative stress caused by the imbalance between antioxidant defenses and production of reactive oxygen species (ROS). The stress can be increased by increasing the generation of ROS and/or reducing the elimination of ROS by antioxidant. As a result, DM arises due to the excessive production of ROS and impairment of the antioxidant defense system. Thus, a compound that inhibits the oxidative stress will be regarded as an antioxidant [23].

According to Al-Zuaidy et al. [23], four major bioactive compounds—isorhamnetin, scopoletin, skimmianine, and melicarpinone—were found in the ethanolic leaf extracts of *M. lunu-ankenda*. The functions of these compounds are briefly described in Table 5. By using the inhibition of the α-glucosidase assay, they found that 40%, 60%, and 80% ethanolic extracts showed significant antidiabetic activity with IC_50_ of 41 μg/mL, 37 μg/mL, and 39 μg/mL, respectively. Their findings also revealed that 60% ethanolic extract showed the highest inhibitory effect against α-glucosidase among the other extracts of different concentrations. Besides, 2,2-diphenyl-1-picrylhydrazyl (DPPH) assay was used to evaluate the radical scavenging potential of the extracts, and they found that all of the extracts of different concentrations demonstrated potent antioxidant effect, with IC_50_ of 48 μg/mL and 53 μg/mL for 60% and 80% ethanolic extracts, respectively. In addition, they also reported the in vivo antidiabetic effect of the extracts in experimented rats. The rats treated with extracts at 400 mg/kg body weight (BW) revealed a noticeable reduction in fasting blood glucose levels compared to diabetic rats, with the suppression in fasting blood glucose level by 62.75%.

George et al. [18] successfully isolated 3,5,4′-trihydroxy-8,3′-dimethoxy-7-(3-methylbut-2-enoxy) flavone, a unique *O*-prenylated flavonoid (OPF) from the petroleum ether extract of *M. lunu-ankenda* leaves. They found this compound significantly reduced the blood glucose level by using an oral glucose tolerance test on overnight fasted, glucose-loaded normal rats. They found that OPF at 10 mg/kg and 25 mg/kg body weight showed a significant reduction in blood glucose levels at 30 min and 90 min after glucose administration, with the activity of OPF at a dose of 10 mg/kg body weight, was taken as the optimum, as dose-dependent glucose-lowering activity was not exhibited for the compound at 25 mg/kg body weight. In neonatal streptozotocin (STZ)-induced diabetic rats, the blood glucose levels reduced (98.1 ± 2.4 mg/dL), and were comparable to control after 20 days of OPF treatment. Moreover, serum biochemical parameters (SGOT, SGPT, ALP, protein and serum insulin levels) in STZ-induced rats were restored to normal levels (91.9 ± 50 IU/L, 23.4 ± 4.1 IU/L, 12.9 ± 1.5 KAU, 7.1 ± 1.0 g/dL, and 1.39 ± 0.1 ng/mL, respectively) when treated with OPF. Their findings also reported that a single dose of OPF at 500 mg/kg body weight did not exhibit any toxic symptoms in mice in acute toxicity study. They also conducted insulin release assay on cultured RIN 5F cells to elucidate the mechanism of OPF for its antidiabetic activity. The mechanism of OPF is briefly described in Table 5.

**Figure 2:**
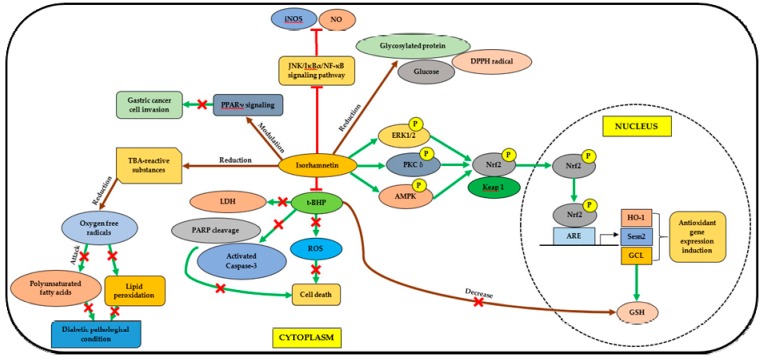
The mechanisms of antidiabetic activity exhibited by isorhamnetin. TBA, thiobarbituric acid; LDH, lactate dehydrogenase; PARP, poly (ADP-ribose) polymerase; ROS, reactive oxygen species; t-BHP, tert-butyl hydroperoxide; PPARγ, peroxisome proliferator-activated receptor gamma; JNK, c-Jun N-terminal kinase; IκBα, nuclear factor of kappa light polypeptide gene enhance in B-cells inhibitor, alpha; NF-κB, nuclear factor kappa-light-chain-enhancer of activated B cells; iNOS, inducible nitric oxide synthase; NO, nitric oxide; DPPH, 1,1-Diphenyl-2-picrylhydrazyl; ERK1/2, extracellular signal-regulated kinase 1 or 2; PKC δ, protein kinase C delta; AMPK, 5′ adenosine monophosphate-activated protein kinase; Nrf2, nuclear factor erythroid 2-related factor 2; Keap1, Kelch-like ECH-associated protein 1; ARE, antioxidant response element; HO-1, hemeoxygenase 1; Sesn2, sestrin2; GCL, glutamate-cysteine ligase; GSH, glutathione. Adapted from Yang et al. [53].

**Figure 3:**
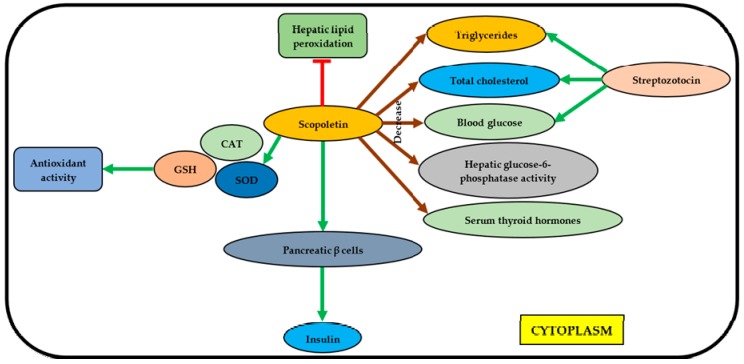
The mechanisms of antidiabetic activity exhibited by scopoletin. GSH, glutathione; CAT, catalase; SOD, superoxide dismutase.

The findings rationalized the use of *M. lunu-ankenda* for diabetic treatment in folk medicine tradition, and suggested that *M. lunu-ankenda* could be used for the development of new generation antidiabetic drugs. Figure 2 and Figure 3 show the mechanisms of antidiabetic activities exhibited by isorhamnetin and scopoletin.

Isorhamnetin (in HepG2 cells) increases the nuclear translocation of Nrf2, decreases cytosolic nuclear factor erythroid 2-related factor 2 (Nrf2) in a dose-dependent manner, and consistently increases antioxidant response element (ARE) activity, and therefore, the induction of antioxidant gene expression such as hemeoxygenase 1 (HO-1), glutamate-cysteine ligase (GCL), and sestrin2 (Sesn2). The increase of glutathione (GSH) levels is suggested to be due to the induction of GCL expression [53]. Isorhamnetin can decrease the levels of glycosylated protein, glucose, and DPPH radicals as well as thiobarbituric acid (TBA)-reactive substances, which result in a reduction of oxygen free radical levels, and thus ameliorate the diabetic pathological condition [54]. The chemical is able to inhibit tert-butyl hydroperoxide (t-BHP)-induced reactive oxygen species (ROS) production and cell death (apoptosis), and reverse GSH depletion, as well as decrease the level of released lactate dehydrogenase (LDH). Furthermore, isorhamnetin was also found to inhibit gastric cancer cell invasion by modulating PPARγ signaling, and found to be suppressing inducible nitric oxide synthase (iNOS) expression and nitric oxide (NO) production in LPS-activated macrophages by inhibiting c-Jun N-terminal kinase (JNK)/nuclear factor of kappa light polypeptide gene enhance in B-cells inhibitor, alpha (IκBα)/(nuclear factor kappa-light-chain-enhancer of activated B cells (NF-κB) signaling pathway [53]. The pathway of Nrf2 nuclear translocation and the induction of expression of antioxidant genes are adapted from Yang et al. [53].

Scopoletin was discovered to inhibit the hepatic lipid peroxidation (LPO) and able to increase the activity of antioxidants including superoxide dismutase (SOD), catalase (CAT) and glutathione (GSH) [56]. Besides, it was also to slightly regenerate pancreatic β cells, thus secreting insulin. Moreover, scopoletin can decrease the activity of glucose-6-phosphatase and the levels of serum thyroid hormones, blood glucose, total cholesterol, and triglycerides, in which the levels of the last three molecules were increased by streptozotocin (STZ) [55,56].

### 4.6. Antifeedant

According to Jagadeesh et al. [26], they found that dictamnine or 4-methoxyfuro[2,3-*b*]quinoline showed 62%, and evolitrine or 4,7-dimethoxyfuro[2,3-*b*]quinoline showed 67% insect-antifeedant activities against agricultural pest tobacco caterpillar IV instar larvae *Spodoptera litura* in non-choice laboratory assay. Nevertheless, the mechanism of antifeedant activity was not reported. 

### 4.7. Anti-Inflammatory

Two major chromene-type compounds isolated from the leaf oil of *M. lunu-ankenda*, evodione, and leptonol were discovered to have moderate anti-inflammatory activity against carrageenan-induced paw edema in rats. In both compounds, evodione was revealed to demonstrate better activity than leptonol with 59.4% and 49% inhibition of inflammatory reaction at 100 mg/kg. This is also true for both compounds at 25 mg/kg and 50 mg/kg, respectively. The positive control (indomethacin) showed 74% inhibition of inflammatory reaction at 10 mg/kg [8].

Lal et al. [27] reported that evolitrine could be an effective anti-inflammatory/immunomodulatory agent, as it effectively inhibited the carrageenan-induced rat paw edema. The percentage of inhibition of carrageenan-induced rat paw edema was found to increase significantly (12%, 57%, 65%, and 78%) as the dose of evolitrine increased (10 mg/kg, 20 mg/kg, 40 mg/kg, and 60 mg/kg, respectively). A carrageenan-induced rat paw model in rat was used because it is the most relevant and widely used model for the prediction of the anti-inflammatory potentials of a drug in acute and chronic inflammatory conditions. They also reported that evolitrine is a more desirable anti-inflammatory agent, as it was found to be as potent as indomethacin (55% inhibition of carrageenan edema at 5 mg/kg), and does not cause gastric irritation.

### 4.8. Antioxidant

Based on Johnson et al. [8], leptonol demonstrated moderate antioxidant activity in a dose-dependent manner in DPPH radical scavenging activity. The inhibitory percentage of DPPH radicals for leptonol is 68.75% at 500 μM. However, evodione showed almost no antioxidant activity (1.36% inhibition), even at the dose of 500 μM. The similar result was also found in superoxide radical scavenging assay for the evaluation of antioxidant activity. They discovered that leptonol showed 64.46% of inhibition at 100 μg/mL, while evodione showed 10.32% of inhibition at the same concentration. The positive control (quercetin) showed 66.87% inhibition at 10 μg/mL. Both evodione and leptonol are the major chromene-type compounds that are found in *M. lunu-ankenda*.

Izzreen and Noriham [57] found that *M. lunu-ankenda* leaf extract, which was incorporated into the cakes and stored for 15 days at room temperature, showed the most potent antioxidant activity compared to formulations they used with leaf extracts from *Polygonum minus* (*kesum*) and *Murraya koenigii* (curry leaves). They found that the PV (peroxide value) in cakes treated with extract from *M. lunu-ankenda* was the lowest (not rancid and acceptable) for 15 days among all of the other plant extracts used (*kesum* and curry leaves). Besides, they found that the TBA (thiobarbituric acid) value in cakes added with extract of *M. lunu-ankenda* was below 1.0 mg/kg^−1^ sample (rancid but acceptable) up to 15 days. The antioxidant activity of extract from *M. lunu-ankenda* was revealed to be comparable to that of synthetic antioxidants (BHA/BHT), although BHA/BHT showed the strongest antioxidant activity throughout the storage period. Thus, it was suggested that the leaf extract of *M. lunu-ankenda* could be used in the food system as a natural antioxidant agent to extend the shelf life of food products. Nevertheless, their study did not determine the phytochemical composition of the leaf extract.

### 4.9. Antipyretic

Leptonol, which was isolated from the leaf oil of *M. lunu-ankenda*, has been shown to have good antipyretic activity with a reduction of 1.06 °F of rat rectal temperature at a dose of 200 mg/kg after 90 min of the administration of the compound, which was comparable to the positive control acetaminophen (−1.80 °F at 200 mg/kg at 90 min). Meanwhile, the antipyretic activity for evodione was relatively low, with the rat rectal temperature reduced by −0.47 °F at 200 mg/kg at 90 min after the administration. The antipyretic activities for both evodione and leptonol were assayed by Baker’s yeast-induced fever test [8]. However, more research is required to understand how both compounds can give rise to the antipyretic activity of *M. lunu-ankenda*.

### 4.10. Antiviral

According to McCormick et al. [24], buchapine and 3-(3-methyl-2-butenyl)-4-[(3-methyl-2-butenyl)oxy]-2(1*H*)-quinolinone were both found to be active against infectious HIV-1, as confirmed in an XTT-tetrazolium assay using human lymphoblastoid (CEM-SS) host cells (EC_50_ of 0.94 μM, IC_50_ of 29 μM and EC_50_ of 1.64 μM, IC_50_ of 26.9 μM, respectively). Besides, both compounds demonstrated inhibitory activity in an HIV-1 reverse transcriptase assay (IC_50_ of 12 μM and 8 μM, respectively). This suggested that they might be potential candidates for anti-HIV agents. However, three furoquinoline alkaloids—roxiamines A, B, and C—were found to be inactive against HIV-1.

### 4.11. Fungicidal

Kumar et al. [30] found that two out of three phenylethanones, 1-[2′,4′-dihydroxy-6′-(3″-methyl-2″-butenyloxy)-5′-(3″-methyl-2″-butenyl)]phenylethanone and l-[2′,4′-dihydroxy-6′-(3″,7″-dimethylocta-2″,6″-dienyloxy)-5′-(3″-methyl-2″-butenyl)]phenylethanone, demonstrated fungicidal activity. They newly isolated these two phenylethanones.

### 4.12. Insecticidal

Based on the study by Ramli et al. [19], the larvicidal bioassay on the crude extracts (dichloromethane and methanol) of *M. lunu-ankenda* leaves revealed that both extracts showed high larvicidal (insecticidal) activity due to their very low LC_50_ values (8.65 μg/mL and 12.63 μg/mL, respectively). The same study also isolated a *p*-coumaric acid derivative, called *p*-*O*-geranylcoumaric acid (3-{4-*O*-(3,7-dimethyl-2,6-octadienyl) phenyl}-2-propenoic acid) with LC_50_ value of more than 100 μg/mL, indicating very weak larvicidal activity. Besides, *p*-coumaric acid was demonstrated to have antibacterial activity as well, which kills bacteria through dual damage mechanisms: significantly increasing the permeability of the bacterial outer and plasma membrane, resulting in the loss of the barrier function and binding to bacterial genomic DNA to inhibit cellular functions, including replication, transcription, and expression [58].

## 5. Conclusions

Due to the increasing microbial resistance and side effects of synthesized drugs or medicine, herbs or medicinal plants are being given more attention than before. In addition, researchers found that the phytochemical compounds that were isolated from the herbs or medicinal plants are relatively safe and effective compared to chemically synthesized drugs or medicines. *Melicope lunu-ankenda* is one of the plant species that has been used in folklore medicinal practices for decades, especially in Asia, including Malaysia. Researchers have isolated various phytochemical compounds from different parts of *M. lunu-ankenda*, and they found that they demonstrated different beneficial biological activities such as analgesic, antidiabetic, cytotoxic, insecticidal, and antiviral activities. These findings not only justify the use of *M. lunu-ankenda* in folk medicine tradition but also indicate that it has potential in food and health industries.

## Figures and Tables

**Table 1:** Main morphological differences between genera *Euodia*, *Tetradium,* and *Melicope.* Adapted from Hartley [5].

Genus	Main Morphological Difference
*Euodia*	Seeds dull and roughened, discharge when follicle dehisces Leaves trifoliolate or unifoliolate Inflorescences axillary
*Tetradium*	Seeds shiny and smooth, remain attached in the dehisced fruit Leaves pinnately compound Inflorescences terminal or terminal and from the axils of the uppermost leaves pair
*Melicope*	Seeds shiny and smooth, remain attached in the dehisced fruit Leaves trifoliolate, unifoliolate or simple Inflorescences axillary, lateral or rarely terminal

**Table 2:** The taxonomy of *M. lunu-ankenda* [9].

Taxonomic Rank	Taxon
Kingdom	Plantae
Phylum	Tracheophyta
Class	Magnoliopsida
Order	Sapindales
Family	*Rutaceae*
Genus	*Melicope*
Species	*Melicope lunu-ankenda* (Gaertn.) T.G. Hartley

**Table 3:** The group, chemical structure, and biological activity of some phytochemical compounds in *M. lunu-ankenda*.

Group	Compound	Chemical Structure	Biological Activity	Ref.
Alkaloids	4-Methoxy-1-methyl-2(1H)-quinolinone	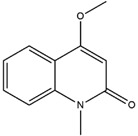	Not reported	[29,33]
	Isolycodolin	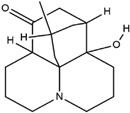	Antibacterial	[28,34]
	3-(3-Methyl-2-butenyl)-4-[(3-methyl-2-butenyl)oxy]-2(1H)-quinolinone	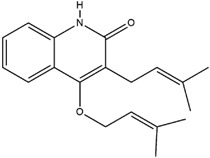	Antiviral	[24]
	Buchapine	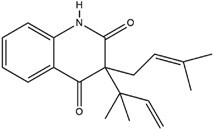	Antiviral	[24]
	Dictamnine (4-Methoxyfuro[2,3-b]quinoline)	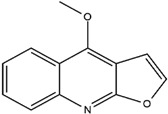	Antifeedant	[25,26]
	Evolitrine (4,7-Dimethoxyfuro[2,3-b]quinoline)	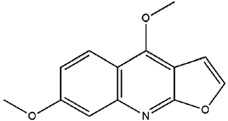	Antifeedant and anti-inflammatory	[25,26,27,29,30,31]
	Kokusaginine	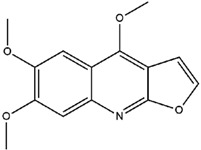	Not reported	[25,30,31,35]
	Melicarpinone	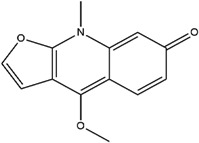	Antidiabetic and anti-inflammatory	[23]
	Roxiamine A	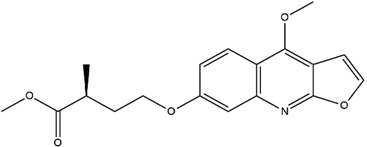	Inactive	[24,36]
	Roxiamine B	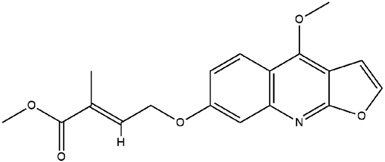	Inactive	[24,37]
	Roxiamine C	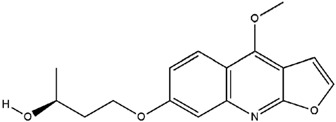	Inactive	[24,38]
	Skimmianine	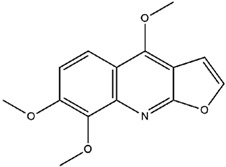	Antidiabetic and anti-inflammatory	[23]
	γ-fagarine	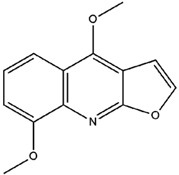	Not reported	[30,39]
Chromenes	Alloevodionol	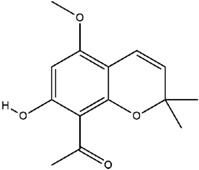	Antibacterial	[28,40]
	Evodione	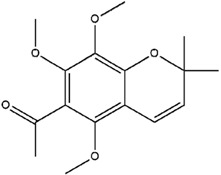	Analgesic, anti-inflammatory, and Antibacterial	[8,28]
	Isoevodionol	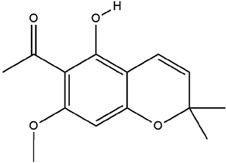	Not reported	[29,41]
	Leptonol	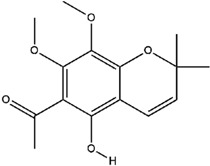	Antipyretic, antioxidant, analgesic, and anti-inflammatory	[8]
	Melilunumane (3,4-Dihydro-3,4-dihydroxy-2,2-dimethyl-2H-1-benzopyran-6-carboxaldehyde)	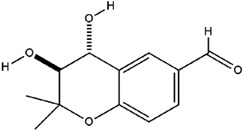	Not reported	[31]
Coumarins	6-Deoxyhaplopinol	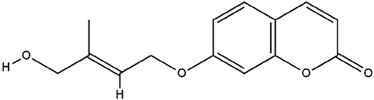	Chemopreventive	[31,42]
	Marmesin	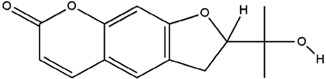	Antiproliferative and chemopreventive	[25,31,43]
	Melilunumarin A (7-(4-Hydroxy-3-methylbutanoxy) coumarin)	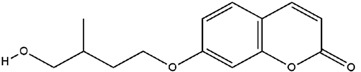	Chemopreventive	[31]
	Melilunumarin B (7-(3-Methyl-4-carboxybutanoxy) coumarin methyl ester)	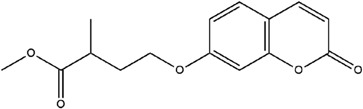	Chemopreventive (Weak)	[31]
	Scopoletin	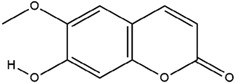	Antidiabetic and antioxidant	[23]
	Umbelliferone	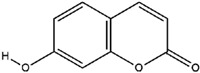	Not reported	[31,44]
	Bergapten	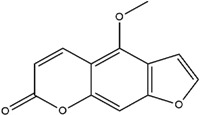	Not reported	[30,45]
Coumaric acids	*p*-*O*-geranylcoumaric acid (3-{4-*O*-(3,7-dimethyl-2,6-octadienyl) phenyl}-2-propenoic acid)	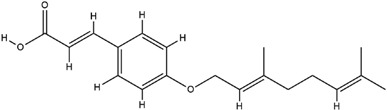	Inactive	[19]
Flavonoids	Isorhamnetin	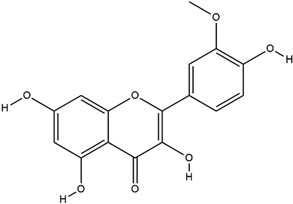	Antidiabetic and antioxidant	[23]
	*O*-prenylated flavonoid (3,5,4′-Trihydroxy-8,3′-dimethoxy-7-(3-methylbut-2-enoxy) flavone)	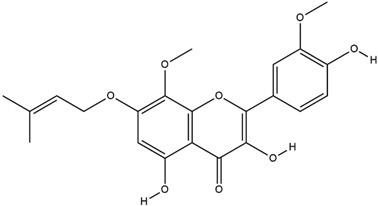	Antidiabetic	[18]
Terpenoids	(E)-β-ocimene	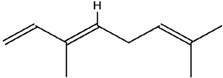	Antibacterial	[28,46]
	Lupeol	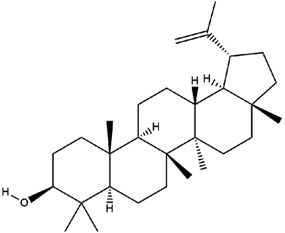	Not reported	[30,47]

**Table 4:** Brief description of functions or mechanisms of extracts and compounds isolated from *M. lunu-ankenda*.

Extract/Compound	Cancer Cell	Function/Mechanism	Ref.
60% ethanolic extract	HepG2	Exhibit cytotoxicity without affecting normal cell line (3t3-L1)	[50]
Melilunumarin A	Raji	Inhibit the TPA-induced Epstein-Barr virus early antigen (EBV-EA) activation	[31]
6-deoxyhaplopinol			
Marmesin	A549	Inhibit cell proliferation Inhibit the TPA-induced Epstein-Barr virus early antigen (EBV-EA) activation	
	KB		
	HUVECs, A549 & H1299	Inhibit VEGF-A-stimulated endothelial cell proliferation Prevent VEGF-A-induced endothelial cell migration and invasion Exhibit anti-angiogenic activity Suppress the expression and secretion of VEGF	[51,52]

**Table 5:** Brief description of functions or mechanisms of compounds isolated from *M. lunu-ankenda* for antidiabetic activity. ARE: antioxidant response element; DPPH: 2,2-diphenyl-1-picrylhydrazyl; iNOS: inducible nitric oxide synthase; NO: nitric oxide; Nrf-2: nuclear factor erythroid 2-related factor 2; ROS: reactive oxygen species; STZ: streptozotocin; t-BHP, tert-butyl hydroperoxide; TBA: thiobarbituric acid.

Compound	Function/Mechanism	Ref.
Ethanolic extract	Inhibit α-glucosidase activity (key enzyme in carbohydrate hydrolysis)	[23]
	Stimulate dose-dependent glucose uptake in both 3t3-L1 and HepG2 cells	[50]
Isorhamnetin	Protect hepatocytes against oxidative stress by activating Nrf2-ARE pathway Block t-BHP induced ROS production and cell death Inhibit invasion and migration of gastric cancer cells Inhibit the expression of iNOS and the production of NO in LPS-activated macrophages	[53]
	Reduce the levels of DPPH radical, serum glucose and glycosylated proteins	[54]
	Protect serum and tissue mitochondria from lipid peroxidation Reduce TBA-reactive substance levels	
	Exhibit antioxidant activity in STZ-induced diabetic rats	
Scopoletin	Reduce the levels of blood glucose and lipid in STZ-induced diabetic rats Regenerate pancreatic-β cells slightly	[55]
	Reduce hyperthyroid and hyperglycemic conditions without hepatotoxic effects Inhibit hepatic lipid peroxidation Increase the activities of superoxide dismutase, catalase and glutathione Decrease the levels of serum thyroid hormone and glucose Reduce the activity of hepatic glucose-6-phosphatase	[56]
Skimmianine	Exhibit anti-inflammatory activity and in vitro antidiabetic effects	[23]
Melicarpinone	Exhibit anti-inflammatory activity and in vitro antidiabetic effects	
3,5,4′-trihydroxy-8,3′-dimethoxy-7-(3-methylbut-2-enoxy) flavone (OPF)	Induce insulin release by acting on pancreatic β-cells (cultured RIN 5F cells) to reduce blood glucose levels, serum biochemical parameters and diabetic complications in STZ-induced diabetic rats.	[18]
